# A Tobacco Etch Virus Protease with Increased Substrate Tolerance at the P1' position

**DOI:** 10.1371/journal.pone.0067915

**Published:** 2013-06-24

**Authors:** Christian Renicke, Roberta Spadaccini, Christof Taxis

**Affiliations:** 1 Department of Biology/Genetics, Philipps-Universität Marburg, Marburg, Germany; 2 Dipartimento di Scienze e tecnologie, Universita' degli studi del Sannio, Benevento, Italy; Centro Nacional de Biotecnologia - CSIC, Spain

## Abstract

Site-specific proteases are important tools for *in vitro* and *in vivo* cleavage of proteins. They are widely used for diverse applications, like protein purification, assessment of protein–protein interactions or regulation of protein localization, abundance or activity. Here, we report the development of a procedure to select protease variants with altered specificity based on the well-established *Saccharomyces cerevisiae* adenine auxotrophy-dependent red/white colony assay. We applied this method on the tobacco etch virus (TEV) protease to obtain a protease variant with altered substrate specificity at the P1’ Position. *In vivo* experiments with tester substrates showed that the mutated TEV protease still efficiently recognizes the sequence ENLYFQ, but has almost lost all bias for the amino acid at the P1’ Position. Thus, we generated a site-specific protease for synthetic approaches requiring *in vivo* generation of proteins or peptides with a specific N-terminal amino acid.

## Introduction

The tobacco etch virus (TEV) protease is an important enzyme for life science research. Its high specificity and robustness make it ideal for diverse applications. It is used *in vitro* for protein purification and *in vivo* to test for protein–protein interactions, for induced proteolysis and to generate conditional mutants [1-6]. The biological function of the protease is to proteolyse the viral polyprotein into single proteins during tobacco etch virus biogenesis. The canonical recognition sequence of the protease is given as ENLYFQ-G/S, although with low stringency at several positions [7]. Especially glycine or serine at the seventh position (P1' position) of the recognition sequence can be replaced by another amino acid (except proline), after which at least partial substrate proteolysis has been observed [8].

This tolerance at the P1' position is crucial for one of the *in vivo* techniques based on the TEV protease, the TEV protease induced protein instability (TIPI) system. There, the protease is used to cleave a tag called cODC1-TDegF, which is fused to the target protein. This results in the activation of two degradation sequences (degrons) which induce proteasomal degradation of the degrons and the target [9]. After proteolysis, the degron called TDegF releases an N-degron, which is a destabilizing amino acid exposed at the amino-terminus of a protein [5]. In *Saccharomyces cerevisiae*, 12 of the 20 fundamental amino acids are classified as destabilizing if exposed at the amino-terminus of a protein. They are either directly recognized by the ubiquitin-protein ligase Ubr1 (primary destabilizing amino acids arginine, phenylalanine, leucine, isoleucine, histidine, tyrosine, tryptophan, lysine) or after one or two enzymatic modifications (secondary destabilizing amino acids aspartate, glutamate and tertiary destabilizing amino acids glutamine, asparagine). An accessible lysine residue results in polyubiquitylation of the substrate and subsequently in degradation by the 26S proteasome [10]. Recently, it was found that some amino acids originally considered as stabilizing residues become destabilizing upon acetylation at the α-amino group of their N-terminal residues (methionine, alanine, valine, serine, threonine, cysteine). However, this modification of the N-terminal amino acid takes place only if the second amino acid is not a basic one. Acetylated amino acids are recognized and polyubiquitylated by the ubiquitin-protein ligase Doa10 resulting in proteasomal degradation [11].

The second degron activated by TEV protease proteolysis of the cODC1-TDegF tag is the C-degron cODC1, which is a synthetic degron based on the features of the C-terminal degron of murine ornithine decarboxylase (cODC). Two features are essential for the activity of the synthetic degron: a 37 amino acid-long unstructured peptide at the very carboxy-terminus of a protein and a cysteine-alanine motif, which has to be present roughly in the middle of this unstructured region. This degron is directly recognized and degraded by the proteasome, without the involvement of polyubiquitylation [12]. The cODC1 degron was fused N-terminally to the TDegF degron to engineer a degradation tag with two degrons that protect each other from proteasomal degradation. This bidirectional degradation tag can be placed internally or at either terminus of the target protein. Proteasomal degradation is activated in either case by a single cleavage step by the TEV protease [9].

**Figure 1:**
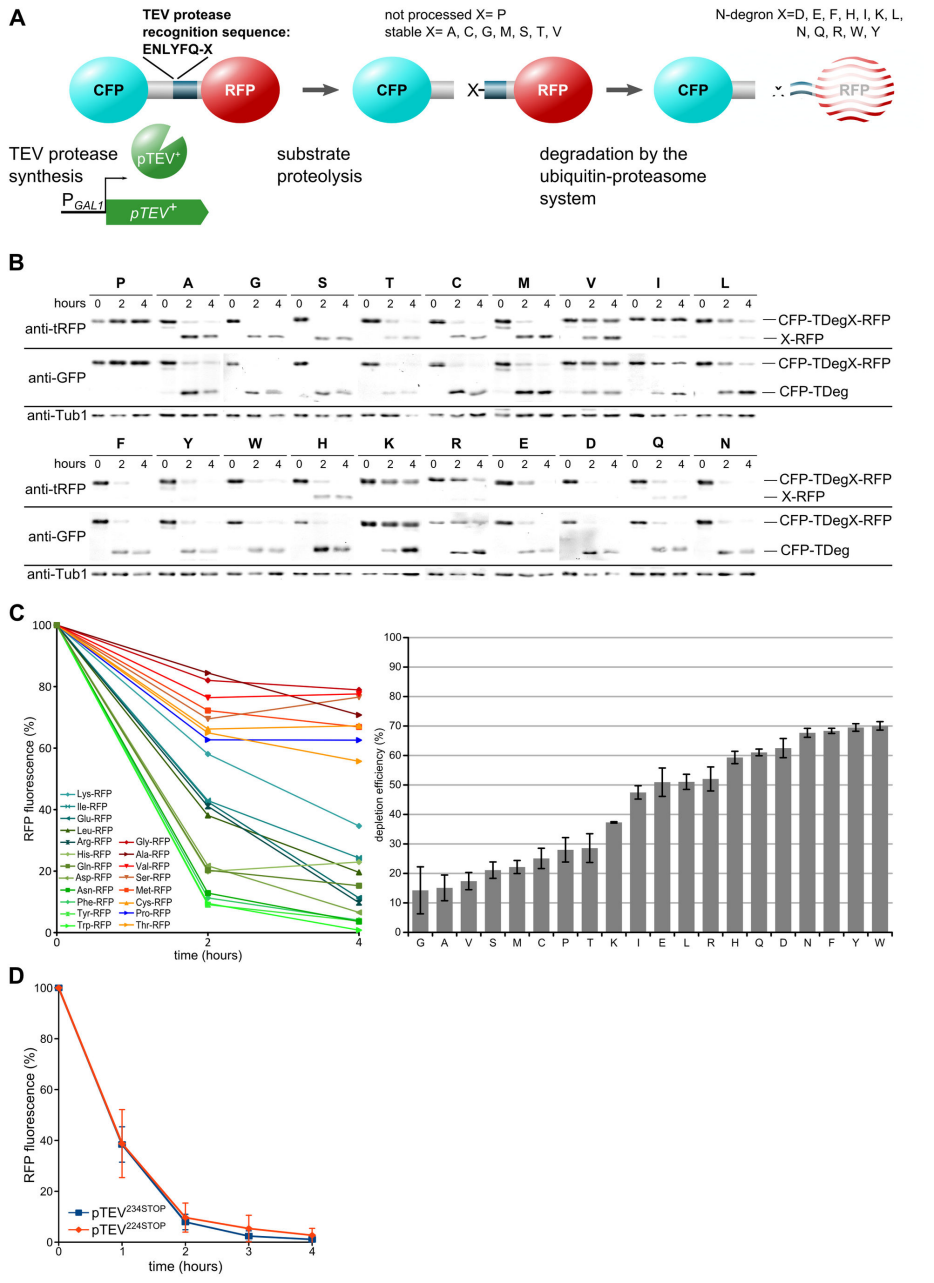
Activation of the N-degron is rate limiting during substrate depletion by the TIPI system. **A**) TIPI efficiency is influenced by three factors, synthesis of the TEV protease by the galactose-inducible *GAL1* promoter, proteolysis of the recognition sequence, and degradation of the target protein by the ubiquitin-proteasome system. A reporter protein consisting of two fluorescent proteins (cyan and red) fused together by the TDegX sequence containing the TEV protease recognition sequence (X= amino acid at position P1') and the N-degron sequence. Please note that we follow the original classification of stabilizing and destabilizing residues without considering N-degrons that are produced by N-acetylation. In our constructs, histidine follows X, which prevents acetylation of X in case of Met, Ala, Ser, Cys, Thr, and Val. **B**) *In vivo* analysis of the P1' specificity of the pTEV^+^ protease. Processing of the tester constructs CFP-TDegX-RFP (plasmid based) was observed after induction of pTEV^+^ protease production (P_*GAL1*_ *-pTEV* ^*+*^ in strain YCT1169) by addition of galactose (2% final concentration). Total cell extracts were fractionated by SDS-polyacrylamide electrophoresis, followed by immunoblotting with antibodies directed against GFP, tRFP and Tub1 (loading control). **C**) Quantification of X-RFP depletion. RFP fluorescence (same constructs as in B) was measured by a fluorimeter after induction of pTEV^+^ protease synthesis (left graph) and the depletion efficiency of the different substrates was calculated (right graph). Curves are mean values of at least four measurements, normalized to initial RFP fluorescence. Depletion efficiency is represented by the area above each curve (error bars: SEM). **D**) C-terminal truncation of the TEV protease at position 224 does not influence its activity. The abundance of the tester substrate CFP-TDegF-RFP was followed over time after expression of different *pTEV protease* variants by fluorimeter measurements (conditions as in C). The plasmid pDS7 was used to express the substrate in yeast strains YCT1243 and YCT1244; error bars represent standard deviation; each construct was measured at least five times.

Overall, three characteristics are important for TIPI: TEV protease production which depends mainly on the expression strength of the promoter chosen for protease production; substrate proteolysis by the TEV protease, which is influenced by protease-substrate interaction and recognition of the cleavage sequence, and the destabilization strength of the activated degron (Figure 1A). During the initial development of the method, the proteolysis rate of the substrate by the TEV protease has been increased by fusing the interacting domains of p14 and SF3B155^381-424^ to the protease and the substrate, respectively. Furthermore, shortening of the protease removed a TEV protease recognition sequence present at the C-terminus that reduces activity of the full length protease by competitive inhibition [5,13]. This engineered variant of the TEV protease has been named pTEV^+^ protease. A systematic test of all amino acids at the P1' position of the recognition sequence has not been undertaken in the context of the TIPI system for the shortened TEV protease, although this position influences both the proteolysis rate and the strength of the N-degron. Work in bacteria has shown that arginine, which is the strongest N-degron [14], decreases substrate proteolysis by the TEV protease considerably if present at the P1' position [8]. Recently, random mutagenesis followed by a screen was performed to find a TEV protease variant with efficient proteolysis of the recognition sequence ENLYFQ-D [15], which is cleaved by the TEV protease *in vitro* and *in vivo* with intermediate to high efficiency, depending on the experimental conditions [5,8]. The TEV protease variant obtained by this screen was found to be less active against the recognition sequence ENLYFQ-S and showed slightly increased activity towards ENLYFQ-D [15].

A powerful genetic technique, which could be used to screen in yeast for TEV protease variants with specific properties, is the adenine auxotrophy-based red/white colony assay. Generation of a red pigment in the yeast vacuole can be observed visually in yeast colonies in this assay if the N-succinyl-5-aminoimidazole-4-carboxamide ribotide synthetase (Ade1) or the phosphoribosylaminoimidazole carboxylase (Ade2) is not functional. This screen has been used among many other purposes to identify genetic interactions, study chromosome stability, or examine protein function [16-18]. In most cases, assays used a procedure that indicated the presence of the *ADE1* or *ADE2* gene. However, we reasoned that it should be possible to use this assay in the context of the TIPI system to visualize protein stability, as it has been done for another degron as well [19].

Here, we report a detailed analysis of the influence of different amino acids at the P1' position of the recognition sequence on the processivity of a shortened TEV protease variant. Furthermore, we describe the development of an Ade2-based assay that can be used to search for protease mutants with changed substrate preference or for conditions that influence substrate proteolysis. Combining this procedure with random mutagenesis, we obtained a TEV protease variant with increased *in vivo* processivity of recognition sequences containing large, branched and positively charged amino acids at the P1' position. This TEV protease mutant showed almost no P1' position preference within the context of the TIPI system and might be a valuable tool for other experiments requiring site-directed proteolysis as well.

## Results

### *In vivo* proteolysis of substrates with different recognition sequences by the pTEV^+^ protease

First, we wanted to know to which extent different amino acids at the P1' position of the recognition sequence influence proteolytic activity of the pTEV^+^ protease, which has higher processivity due to removal of the last 8 amino acids and increased substrate affinity by the p14-SF3B155^381-424^ domains. We expressed tester substrates (cyan fluorescent protein (CFP)-TDegX-red fluorescent protein(RFP); X = amino acid at the P1' position) containing all 20 fundamental amino acids at the P1' position in yeast cells and followed their proteolysis after induction of pTEV^+^ protease synthesis using the galactose-inducible *GAL1* promoter. We observed that only proline was not processed at all, presence of the other amino acids at this position led to complete or partial proteolysis. Such incomplete proteolysis was found for the constructs with arginine, isoleucine, leucine, lysine, or valine at the P1' position. In these cases full length CFP-TDegX-RFP was detected four hours after induction of pTEV^+^ protease production, whereas no full length tester substrate was observable for the other constructs. In general, a higher degree of proteolysis was obtained with smaller amino acids at the P1' position compared to larger amino acids, aromatic to aliphatic and negatively to positively charged ones (Figure 1B). The pattern we found is in good agreement with the data obtained with full-length TEV protease without a domain to increase substrate affinity [8].

To measure the combined effect of proteolytic efficiency and destabilization strength, we quantified the stability of the RFP part of the tester substrates after induction of pTEV^+^ protease production. This revealed that amino acids that are classified as stabilizing according to the "classical" view [10] showed only a slight decrease of RFP fluorescence. A similar decrease was also observed for the uncleavable CFP-TDegP-RFP substrate (Figure 1C), which suggests declined tester substrate synthesis at later time points. Due to the presence of histidine at the P2' position, no acetylation and subsequent destabilization via the Doa10 pathway is expected for these amino acids. Exposure of a destabilizing residue at the N-terminus resulted in complete depletion of X-RFP in most cases. To measure differences between the constructs, we calculated depletion efficiencies that reflect how fast a substrate is processed and degraded. For most destabilizing amino acids at the P1' position, depletion efficiencies between 60 and 70% were reached (Figure 1C). Remarkably, the tertiary N-degron asparagine at the P1' position was found to be among the residues with highest depletion efficiency (Figure 1C), although the transformation of this amino acid into an N-degron requires two additional modifications after TEV protease cleavage [10]. Furthermore, we observed that constructs with lysine, isoleucine, leucine, glutamate, and arginine at the P1' position, which were processed by the pTEV^+^ protease with low efficiency (Figure 1B), exhibited depletion efficiencies around 50% or below (Figure 1C). The correlation between TEV protease cleavage and depletion efficiency as well as the high depletion efficiency of asparagine suggest that the rate-limiting step during substrate depletion is proteolysis by the TEV protease. Moreover, our analysis showed that the strongest N-degron (arginine) at the P1' position is severely disfavored for cleavage by the TEV protease. Next, we tested whether further shortening of the TEV protease (stop codon at position 224 of the TEV protease sequence compared to stop codon at position 234) would increase processivity of the protease. However, we found no difference between the two proteases towards TDegF containing substrates (Figure 1D) as well as substrates with TDegR and TDegP (data not shown).

### Screen for a TEV protease variant with improved recognition of arginine at the P1' position

**Figure 2:**
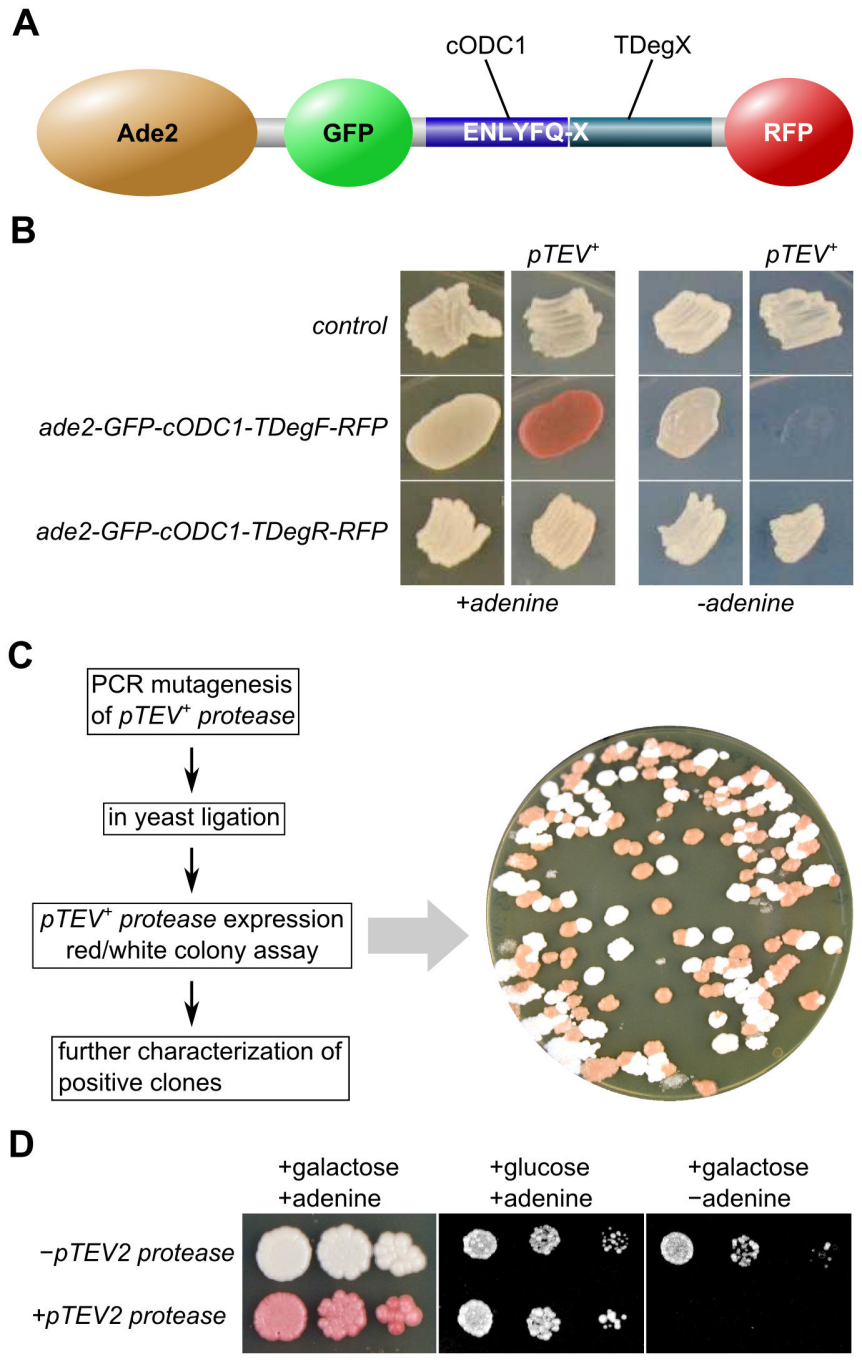
Generation of a TEV protease that cleaves efficiently the recognition sequence ENLYFQ-R. **A**) Scheme of the construct used for the screening procedure: The bidirectional degron module GFP-cODC1-TDegX-RFP (X = F or R) was fused to the phosphoribosylaminoimidazole carboxylase Ade2. Cleavage by the TEV protease leads to activation of the C-degron cODC1 and the N-degron TDegX resulting in proteasomal degradation of Ade2-GFP-cODC1 as well as TDegX-RFP. **B**) Test for adenine biosynthesis in cells bearing different degron constructs fused chromosomally to *ADE2*. The yeast strains (ESM356-1, YCT1266, and YCR8) were grown in patches on solid media (YPD, YP+galactose, yeast nitrogen base + 2% glucose, and yeast nitrogen base + 2% galactose; from left to right). **C**) Scheme illustrating the mutagenesis and selection procedure to obtain a TEV protease which efficiently processes the recognition sequence ENLYFQ-R (left side). The plate is an example to show the difference in color of clones with efficient proteolysis of ENLYFQ-R (red colonies) and clones with insufficient proteolysis (white colonies). Please note that the high degree of red colonies was obtained because the R345G mutant was generated already in the first round of mutagenesis and enriched in subsequent rounds. **D**) Expression of *pTEV2 protease* (plasmid-based, R345G mutant) using the *GAL1* promoter induces the adenine auxotrophy phenotype in *ade2-GFP-cODC1-TDegR-RFP* cells (YCR6). Serial dilutions (1:10) were grown on solid media as in B.

To increase the proteolysis rate of a TEV protease recognition sequence with arginine at the P1' position, we set up a screening procedure which allowed us to select for a TEV protease with efficient proteolysis of the recognition sequence ENLYFQ-R. We fused two variants of the bidirectional degron green fluorescent protein (GFP)-cODC1-TDegX-RFP (X=F, R) to the Ade2 enzyme which is necessary to produce adenine (Figure 2A). Upon induction of pTEV^+^ protease synthesis in these strains, the cells containing the phenylalanine construct showed an adenine auxotrophy phenotype. The cells were red on adenine-containing medium and unable to grow on adenine-free medium, whereas control cells or cells bearing the arginine construct were adenine prototroph (Figure 2B). This demonstrates that only efficient proteolysis of the degron construct induces depletion of the modified Ade2 and evokes the adenine auxotrophy phenotype. This clear Ade^−^ phenotype in cells bearing the TDegF construct indicated that screening for a TEV protease that efficiently processes the recognition sequence ENLYFQ-R might be possible.

We used PCR-based random mutagenesis and homologous recombination in yeast to generate a pool of plasmids containing *pTEV+ protease* mutants in the *ade2-GFP-cODC1-TDegR-RFP* strain. The plasmids of transformants which showed a red colony phenotype on TEV protease production-inducing galactose plates were rescued from yeast into *Escherichia coli*, retransformed and tested with a patch assay as well as immunoblotting for efficient Ade2 depletion (Figure 2C and data not shown). Plasmids of confirmed transformants were sequenced and used as template for further rounds of mutagenesis, in total about 1200 clones were screened. All of the tested *pTEV* ^*+*^ *protease* alleles, obtained from the last round, encoded for a protein with a single amino acid exchange. In these mutants, the arginine at position 345, which corresponds to R203 in the TEV protease sequence, was changed to glycine. The *ade2-GFP-cODC1-TDegR-RFP* strain transformed with a plasmid containing the R345G mutant was subjected to a serial dilution growth assay. The strain showed adenine auxotrophy upon production of the mutated protease, as expected (Figure 2D). This demonstrated that the chosen strategy to obtain a pTEV protease with efficient proteolysis of the recognition sequence ENLYFQ-R was successful. Subsequently, we will refer to this mutant version as pTEV2 protease.

### P1'-dependent substrate selectivity of the pTEV2 protease

**Figure 3:**
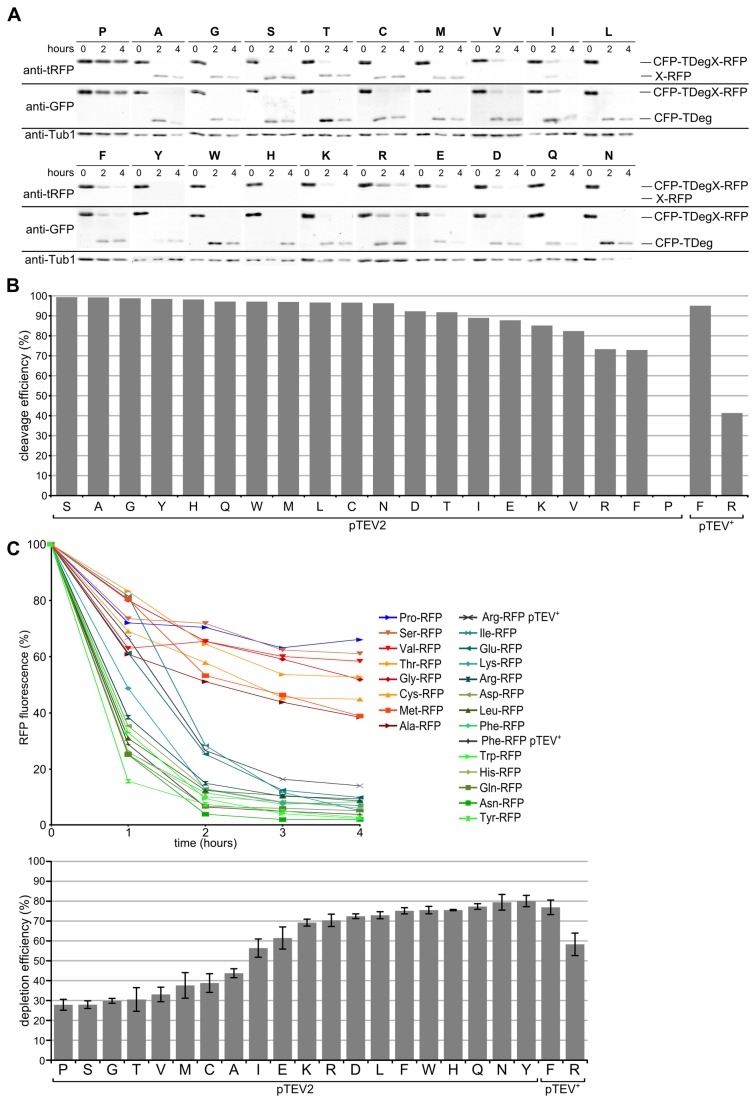
*In vivo* analysis of the P1' Specificity of the pTEV2 protease. **A**) Processing of the tester constructs CFP-TDegX-RFP (plasmid encoded) was observed after induction of pTEV2 protease production (P_*GAL1*_ *-pTEV2* in yeast strain YCR56). Conditions as in Figure 1B. **B**) Quantification of the P1' Specificity of the pTEV2 protease. Decrease of full length tester construct after two hours was normalized to initial values and relative efficiency normalized to proline was calculated (cleavage efficiency = ([X]_2h_/[Pro]_2h_×100-100) ×(−1)), assuming that the recognition sequence with proline at the P1’ Position is not cleaved at all. For each construct two immunoblotting experiments were quantified. Values for constructs with Arg and Phe at the P1’ Position cleaved by the pTEV^+^ protease obtained at the same time are shown as reference. Yeast strains YCR56 (pTEV2 protease production) or YCT1169 (pTEV^+^ protease production) harboring plasmid-based constructs were used for the measurements. **C**) Quantification of X-RFP depletion. The RFP fluorescence was analyzed by fluorimeter measurements after induction of pTEV2 protease synthesis (upper graph, conditions as in Figure 1C) and the depletion efficiency was calculated (error bars: SEM of at least three experiments). Same constructs as in B. The difference between the arginine construct cleaved by pTEV2 and pTEV^+^ protease is very significant (unpaired t test; p = 0.007).

To test whether the pTEV2 protease has an altered substrate preference, we assessed the efficiency of proteolysis of all 20 fundamental amino acids at the position P1'. Again, we used the CFP-TDegX-RFP constructs to follow proteolysis. We found that all recognition sequences with amino acids other than proline at the P1' position were processed efficiently, most constructs were completely processed two hours after induction of pTEV2 synthesis. Substrates with aspartate, glutamate, isoleucine, threonine, and valine showed residual amounts of the full length tester construct after two hours, arginine and phenylalanine also after four hours (Figure 3A). In comparison to the results obtained with the pTEV^+^ protease, constructs with arginine, isoleucine, leucine, lysine or valine at the P1' position were cleaved more efficiently by the pTEV2 protease, indicating that the pTEV2 protease has lost almost all preference for the amino acid at the P1' position. The arginine-containing construct was moderately better cleaved by the pTEV2 protease, whereas the cleavage of the phenylalanine-containing construct was somewhat decreased (Figure 3B). Overall, we found that exchange of a single amino acid in the TEV protease resulted in improved proteolysis of substrates with aliphatic or positively charged amino acids at the P1' position of the TEV recognition sequence *in vivo*.

Moreover, we were interested to follow the fate of the X-RFP part upon production of the pTEV2 protease. We measured the RFP fluorescence of all 20 constructs and found no change in behavior for tester substrates bearing proline or stabilizing amino acids at the P1' position. However, several constructs containing destabilizing residues were depleted much faster upon induction of pTEV2 protease synthesis; their depletion rates were now much more similar to each other. Tyrosine or asparagine at the P1' position induced fastest depletion, whereas substrates with glutamate or isoleucine were slowest. Constructs with the other destabilizing amino acids induced efficient depletion within two to three hours, especially leucine, lysine and arginine were improved considerably (Figure 3C). Our measurements with the CFP-TDegX-RFP substrates revealed that the pTEV2 protease allows generation of some N-degrons with much higher efficiency.

**Figure 4:**
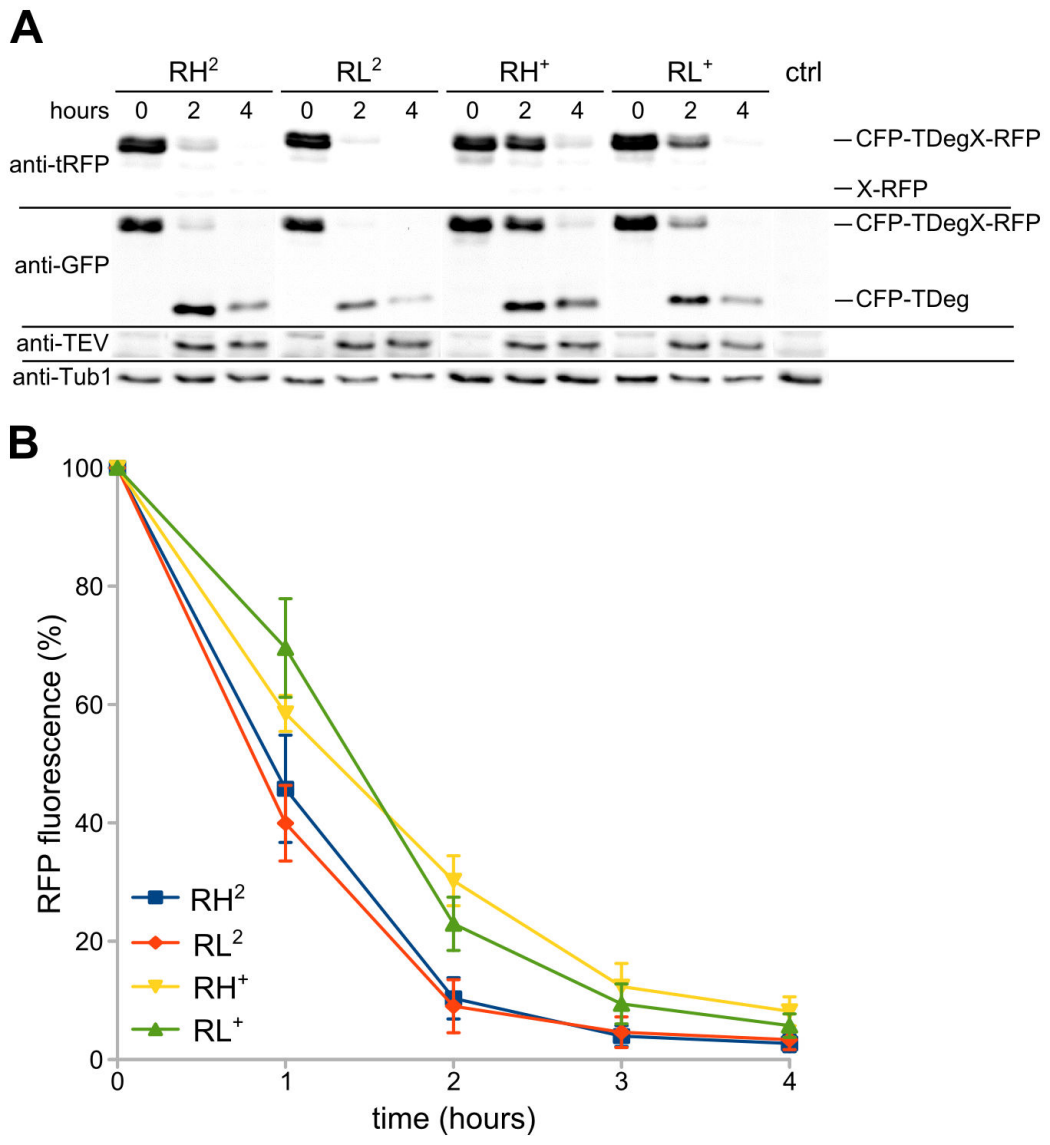
Influence of the P2' residue on substrate degradation. **A**) Analysis of tester construct proteolysis and depletion as well as TEV protease production by immunoblotting. Tester constructs (plasmid based): CFP-TDegXY-RFP, XY=RH, RL, X corresponds to the P1' position, Y to the P2' position; proteases: pTEV^+^ (yeast strain YCT1169), pTEV2 (YCR56). Conditions as in Figure 1B; antibodies directed against tRFP, GFP, TEV, and Tub1 (loading control) were used to obtain the immunoblot. **B**) The RFP fluorescence of the tester constructs CFP-TDegXY-RFP was followed over time after induction of TEV protease synthesis by fluorimeter measurements (three measurements for each construct; error bars indicate the standard error of the mean; same constructs as in A).

Structural analysis of Ubr1 has revealed that a leucine instead of a histidine at the P2' position is favored for recognition of type 1 substrates by the UBR box present in Ubr1 [20]. Therefore, we assessed whether we could further improve depletion efficiency of the construct with arginine at the P1' position by a change of the P2' position. The TEV protease recognition sequence in the CFP-TDegR-RFP tester substrate was changed from ENLYFQ-R**H** to ENLYFQ-R**L** and substrate behavior after induction of protease production was measured. First, we analyzed proteolysis of the RH and RL constructs by both proteases, but did not find a striking difference. Furthermore, the experiment showed that increased proteolysis of the RH and RL constructs by the pTEV2 protease is not due to increased protease production, as protein levels were comparable for both proteases (Figure 4A). Then, we measured depletion of the RFP part of the construct. Again, we did not observe a significant change in depletion of the RL-containing constructs upon proteolysis of the substrates by the pTEV^+^ or pTEV2 protease (Figure 4B). These results strengthen the view that proteolysis by the TEV protease is the rate limiting step during substrate degradation by the TIPI system. The only exceptions might be substrates with glutamate or isoleucine at the P1' position, which are cleaved better by the pTEV2 protease than arginine or phenylalanine-containing substrates (Figure 3B), but which showed a lower depletion efficiency (Figure 3C). Indeed, glutamate and isoleucine have been categorized as the weakest N-degrons [14].

### Predicted structural impact of the R203G mutation

**Figure 5:**
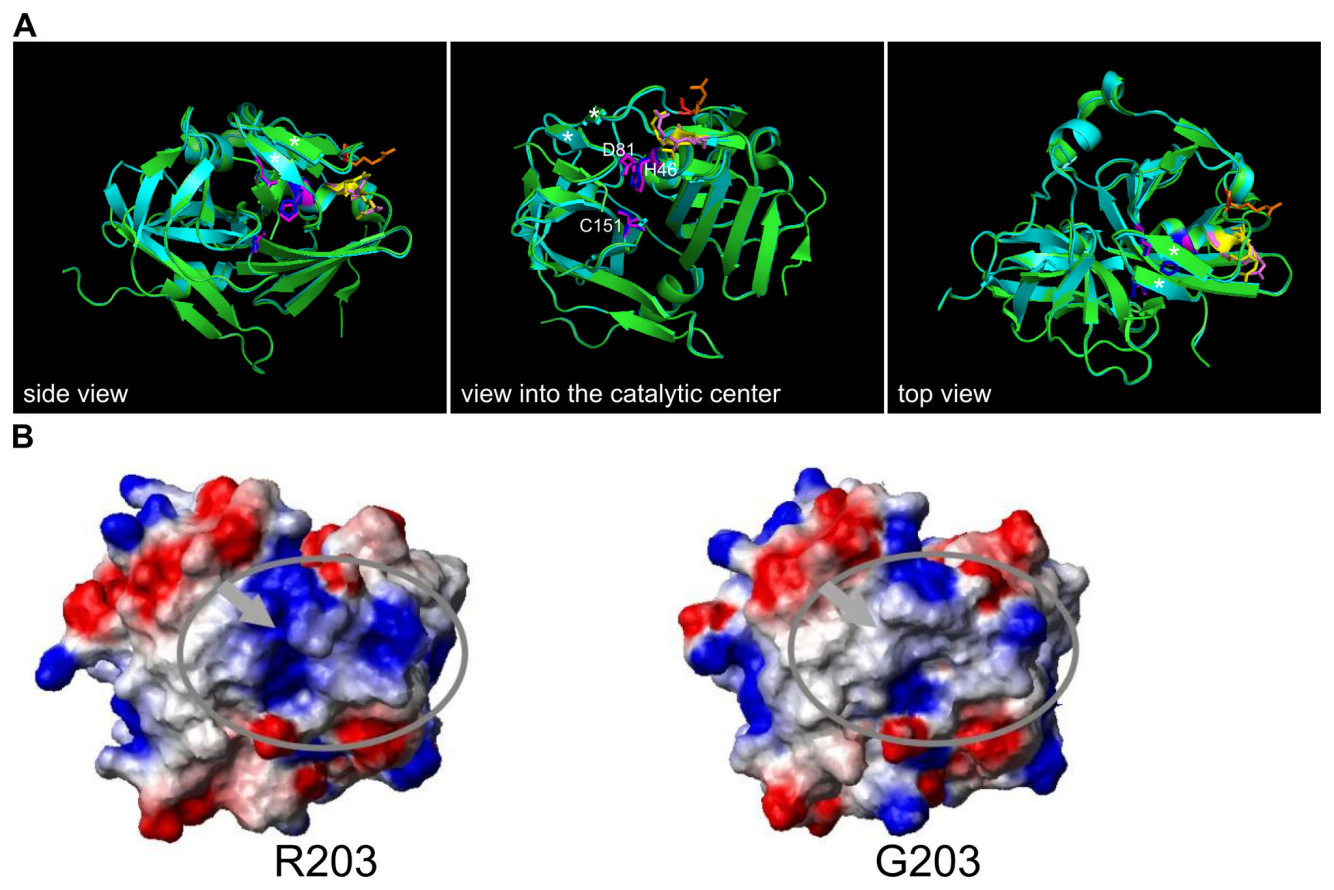
Structural comparison of the TEV protease with the R203G mutant. **A**) Ribbon structure of the TEV protease (green) was overlaid with the mutant (dark cyan). The structure of the R203G mutant, which corresponds to R345G in the pTEV^+^ protease, was obtained by homology modeling using an x-ray structure of the TEV protease as template. Views from three different sides are shown. The residues of the catalytic triad H46, D81, and C151 are indicated (TEV protease: blue; mutant: magenta). The two arginine residues close to the catalytic center (R49, R50) are shown in yellow (TEV protease) and light magenta (G203 mutant). The R203 residue is shown in orange, the G203 in red. The two β-sheets, which are mentioned in the text that close the catalytic center are marked by asterisks. **B**) Surface charge distribution of the TEV protease compared to the R203G mutant. Surface charges were calculated using the software package MolMol. Positive charge is represented by blue color, negative charge by red color.

Finally, we were interested to know whether the R203G mutation has a structural impact on the TEV protease that could explain the changes in substrate preferences. We generated a homology model of the R203G mutant using the published structure of the TEV protease [21]. This revealed no obvious difference within the structure. The mutated residue R203 is located quite far from the catalytic center; it is part of a loop near the C-terminus connecting two beta-sheets with the core of the protease. These two beta-sheets are part of a lid-like structure which is closing the catalytic grove. Subsequently, we performed molecular docking of peptides containing different recognition sequences with the TEV protease and the R203G mutant, but no striking differences were observed concerning binding of the peptides or hydrogen bond formation to residues forming the catalytic center (data not shown). However, we noticed that two arginines (R49, R50) are located between the catalytic center and R203 (Figure 5A). Together, these three residues might create a positively charged surface patch, whereas the R203G mutant would decrease the charge in this area. Indeed, electrostatic surface calculations predicted that the positive charge is reduced in the mutant in this area (Figure 5B), which might allow easier access of a substrate with a positively charged amino acid at the P1' position to the catalytic center.

## Discussion

Here, we studied in detail the usability of the TEV protease as a tool to generate N-degrons for protein destabilization. We found that substrates with the aromatic amino acids phenylalanine, tyrosine and tryptophan or the tertiary N-degron glutamine at the P1' position of the recognition sequence show quickest depletion kinetics among the 20 tested substrates. Furthermore, we present a screening procedure depending on the destabilization of Ade2. This allowed us to select a TEV protease variant that showed, within the context of the TIPI system, a higher *in vivo* processivity of branched aliphatic and positively charged amino acids at position P1'. Structural analysis suggested that better accessibility of the active site might be responsible for the increased substrate tolerance of the mutated TEV protease. This TEV protease variant might be useful to generate peptides or proteins carrying specific amino acids at the N-terminus by site-specific proteolysis.

**Figure 6:**
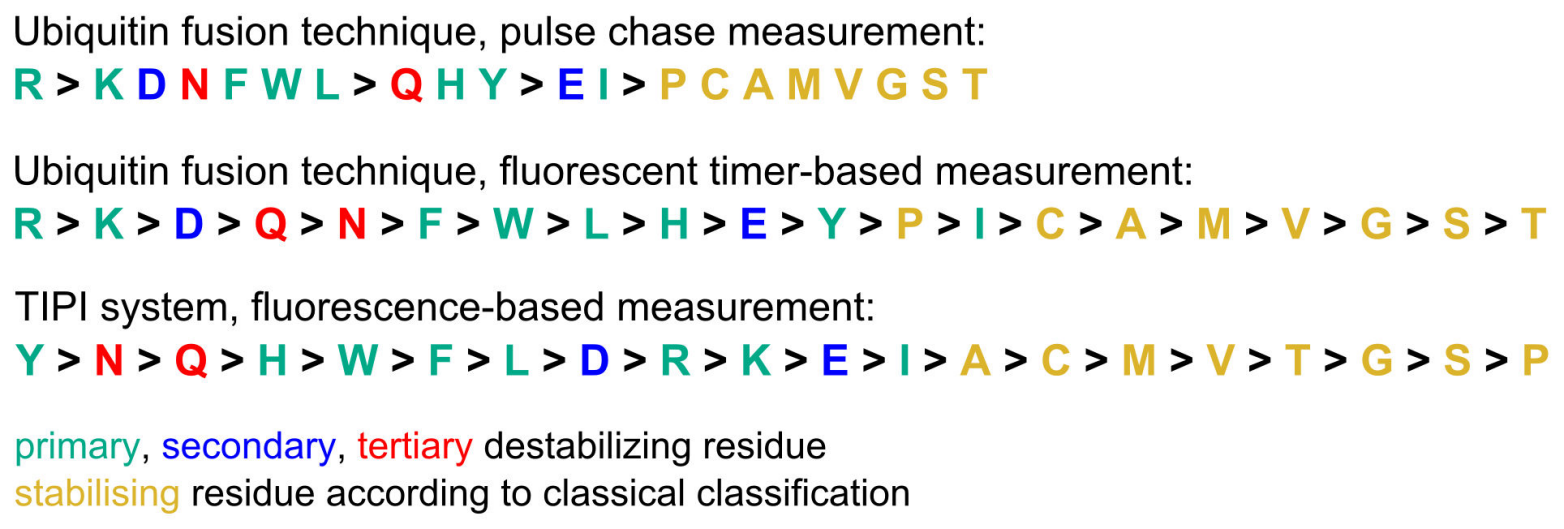
Comparison of the apparent N-degron strength generated and measured by different methods. Apparent N-degron strength ordered from high to low destabilizing activity. Pulse chase data were obtained by Bachmaier et al., 1989, fluorescent timer-based measurements by Khmaelinski et al., 2012, fluorescence-based measurements during this study. A color code indicates whether an amino acid is a primary (dark cyan), secondary (blue) or tertiary (red) destabilizing residue at the amino-terminus of a protein, or if it is stabilizing (gold) in the absence of N-acetylation.

It is interesting to compare the apparent strength of N-degrons generated by the TIPI system or the ubiquitin fusion technique [22]. The same amino acids seem to destabilize quite differently (Figure 6), depending on the method used to measure the half life and the way the N-degron is generated. A possible explanation is that in one or both of the methods the rate-limiting step is not the recognition of the N-degron but its generation. Such a view is supported by the observation that Ubr1 binds peptides having arginine or phenylalanine at the N-terminus with the same affinity *in vitro* [23] as well as by our observation that changing the amino acid at the P2' position to leucine, which increases the affinity of the active N-degron with Ubr1, does not enhance tester protein depletion. Moreover, the differences in depletion efficiency we found for the tester substrates with destabilizing amino acids activated by the pTEV2 protease were not very pronounced and can be explained at least in part by differences in cleavage efficiency. This is best seen for the amino acids glutamate and glutamine. No matter if the ubiquitin fusion technique or the TIPI system is used, glutamine always appears to be the stronger N-degron over glutamate (Figure 6), although glutamine needs to be converted to glutamate before arginylation creates the species recognized by Ubr1. The TIPI system makes it possible to detect the relation between cleavage and degradation efficiency, as the uncleaved species is stable, whereas the ubiquitin fusion technique destabilizes the uncleaved species via the ubiquitin fusion degradation pathway [24].

Notably, shortening of the TEV protease and addition of the p14-SF3B155^381-424^ interaction domains did not change the specificity of the TEV protease. The activity gain observed in the variant with shortened C-terminus originates most likely from release of auto-inhibition due to absence of the very C-terminal located TEV protease recognition sequence [13]. But, the increased processivity of the variant with the interaction domains can be assumed to stem from prolonged protease-substrate interaction. The finding that both changes do not influence the selectivity of the TEV protease towards its substrate strengthen the view that size and structure of the catalytic center govern substrate preference of the TEV protease [25].

Unclear is how many amino acids after the autolysis-site 219 have to be present for full activity of the protease. The amino acids from 221 to 235 have intrinsic flexibility and were not found in the x-ray structures [13,21], but at least a few of these residues are essential for TEV protease activity. Removing the C-terminus up to position 219 leads to almost complete loss of proteolytic activity [26,27], whereas truncation after position 224 resulted in a fully active protease *in vivo*. Although highly flexible, these residues might be important for the correct folding of two beta-strands near the C-terminus that form a kind of lid which closes the catalytic center.

Our screen resulted in a pTEV protease variant that is almost insensitive to changes at the P1' position of the recognition sequence within the context of the TIPI system. Our analysis revealed that for the TIPI system, a TEV protease recognition site with tyrosine or glutamine at position P1' induces depletion of the substrate with highest efficiency. However, the latter residue might not be ideal in all circumstances; glutamine is a tertiary N-degron that requires the efficient execution of two additional enzymatic reactions before the N-degron is recognized by Ubr1. These steps might not always be executed efficiently during a developmental process or in all cell types.

Even though the pTEV2 protease has an extended substrate tolerance, we did not find negative effects of high pTEV2 protease production in yeast. Additionally, the modified protease might not only be useful for *in vivo* applications, also *in vitro* applications might benefit from it. Due to the substrate extension, peptides or proteins with a defined N-terminal amino acid might be produced in higher yields and/or using less TEV protease.

Recently, a random mutagenesis-based approach has been undertaken to increase the processivity of the TEV protease towards the recognition sequence ENLYFQ-D, which is cleaved in bacterial cells with moderate efficiency. In this study, three related TEV protease mutants with up to 14 amino acid exchanges were found. These enzymes displayed enhanced activity towards the non-native substrate combined with decreased proteolysis of the canonical recognition sequence ENLYFQ-S [15]. Most of the mutations are quite far from the catalytic center, which suggests that the substrate selectivity of the TEV protease can be influenced by small changes in the whole protein. Interestingly, one of the mutations, which is present in two of the mutants, is near the catalytic center (R50K) and quite close to the arginine 203, which was mutated to a glycine in case of the pTEV2 protease. Although the amino acids present at the P1' position in the two screens are quite different (aspartate versus arginine), it is tempting to speculate that the electrostatic surface in this region of the protease has an important influence on substrate selectivity. Indeed, bioinformatic analysis of the chymotrypsin family of serine proteases, which is related to the family of 3C cysteine proteases the TEV protease belongs to [28], has shown that substrate specificity is conferred by the catalytic cleft and neighboring surface loops that are thought to stabilize the specific fold of the substrate binding pocket [29].

The selection procedure we developed allows in principle to customize substrate selection by any protease that can be expressed in yeast without toxic effects. Several other selection procedures have been developed in bacteria or yeast previously [15,25,30-33]. Compared to these selection procedures, the Ade2-based assay offers two *in vivo* selection methods (growth/non-growth on adenine-free medium or appearance of red color on adenine-containing medium), which allows a certain flexibility in the set-up of the screen. Importantly, no toxic compound has to be added to the cells, as it is the case for yeast methods based on uracil auxotrophy/5-FOA resistance [34]. Yet, the selection procedures using Ura3 and Ade2 could also be combined to screen for two different selection criteria simultaneously. In principle, the screening procedure can also be reversed to search for protease inhibitors or protease-inhibiting peptides. It might be feasible to exchange the TEV protease with another viral protease and use the adenine-based screening to search in yeast cells for compounds that decrease protease activity.

## Materials and Methods

### Yeast strains, growth conditions and plasmids

The *Saccharomyces cerevisiae* strains are derivatives of the S288C strain ESM356 [35]. All strains are listed with their relevant genotypes in Table **1**. Standard preparations of media were used for growth [36]; low-fluorescence medium [37] was used to grow yeast cells for fluorimeter measurements. Yeast strains with chromosomally encoded *ADE2-GFP-cODC1-TDegF-RFP* and *ADE2-GFP-cODC1-TDegR-RFP* were constructed using PCR products [38] obtained with pCT314 and pCR20 as template, respectively. Yeast transformations with plasmids and PCR products were performed using the lithium acetate method [39].

**Table 1:** Yeast strains used in this study.

Name	Genotype	Source
ESM356-1	*MATa ura3-53 leu2*Δ*1 his3*Δ*200 trp1*Δ*63*	[35]
YCT1169	ESM356 *ura3::P*_*GAL1*_ *-p14* ^*D122Y*^ *-TEV* ^*234STOP*^ *::kanMX*	[5]
YCT1243	ESM356 *ura3::P*_*GAL1*_ *-p14* ^*D122Y*^ *-myc-TEV* ^*234STOP*^ *::kanMX*	This study
YCT1244	ESM356 *ura3::P*_*GAL1*_ *-p14* ^*D122Y*^ *-myc-TEV* ^*224STOP*^ *::kanMX*	[9]
YCR56	ESM356 *ura3::P*_*GAL1*_ *-p14* ^*D122Y*^ *-TEV* ^*R203G 234STOP*^ *::kanMX*	This study
YCT1266	YCT1169 *ADE2-GFP-cODC1-TDegF-mKate::hphNT1*	This study
YCR8	YCT1169 *ADE2-GFP-cODC1-TDegR-mKate::hphNT1*	This study
YCR6	ESM356 *ADE2-GFP-cODC1-TDegR-mKate::hphNT1*	This study

Plasmids were constructed by standard procedures [40], details and sequences of the used vectors are available on request; plasmids are listed in Table **2**. Serial dilution experiments were performed as described [9] with minimal or complex medium supplemented with glucose or galactose. The expression of the pTEV protease variants is repressed on glucose and induced on galactose containing medium. Images were taken with a Canon Powershot A620 digital camera.

**Table 2:** Plasmids used in this study.

Name	Features	Source
pRS414	*TRP1 ARS/CEN*	[41]
pDS7	P_*ADH1*_ *-yeCFP-TDegF-mKATE* in pRS414	[5]
pDS18	P_*ADH1*_ *-yeCFP-TDegM-mKATE* in pRS414	[5]
pDS21-L	P_*ADH1*_ *-yeCFP-TDegL-mKATE* in pRS414	This study
pDS21-N	P_*ADH1*_ *-yeCFP-TDegN-mKATE* in pRS414	This study
pDS21-P	P_*ADH1*_ *-yeCFP-TDegP-mKATE* in pRS414	This study
pDS21-E	P_*ADH1*_ *-yeCFP-TDegE-mKATE* in pRS414	This study
pDS21-K	P_*ADH1*_ *-yeCFP-TDegK-mKATE* in pRS414	This study
pDS21-T	P_*ADH1*_ *-yeCFP-TDegT-mKATE* in pRS414	This study
pDS21-S	P_*ADH1*_ *-yeCFP-TDegS-mKATE* in pRS414	This study
pDS21-G	P_*ADH1*_ *-yeCFP-TDegG-mKATE* in pRS414	This study
pDS21-Y	P_*ADH1*_ *-yeCFP-TDegY-mKATE* in pRS414	This study
pDS21-C	P_*ADH1*_ *-yeCFP-TDegC-mKATE* in pRS414	This study
pDS21-I	P_*ADH1*_ *-yeCFP-TDegI-mKATE* in pRS414	This study
pDS21-R	P_*ADH1*_ *-yeCFP-TDegR-mKATE* in pRS414	This study
pDS21-W	P_*ADH1*_ *-yeCFP-TDegW-mKATE* in pRS414	This study
pDS21-D	P_*ADH1*_ *-yeCFP-TDegD-mKATE* in pRS414	This study
pDS31	P_*ADH1*_ *-yeCFP-TDegH-mKATE* in pRS414	This study
pDS33	P_*ADH1*_ *-yeCFP-TDegV-mKATE* in pRS414	This study
pDS30	P_*ADH1*_ *-yeCFP-TDegA-mKATE* in pRS414	This study
pDS32	P_*ADH1*_ *-yeCFP-TDegQ-mKATE* in pRS414	This study
pCR41	P_*ADH1*_ *-yeCFP-TDegRL-mKATE* in pRS414	This study
pRS313	*HIS3 ARS/CEN*	[41]
pCT310	P_*GAL1*_ *-YFP-p14* ^*D122Y*^ *-TEV* ^*234STOP*^ in pRS313	This study
pRS41N	*natNT2 ARS/CEN*	[42]
pCR30X2	P_*GAL1*_ *-GFP-p14* ^*D122Y*^ *-TEV* ^*R203G 234STOP*^ in pRS41N	This study
pFA6a-hphNT1	*hphNT1*	[38]
pCT314	*GFP-cODC1-TDegF-mKate::hphNT1* in pFA6a-hphNT1	[9]
pCR20	*GFP-cODC1-TDegR-mKate::hphNT1* in pFA6a-hphNT1	This study
pDS15	*ura3::kanMX::*P_*GAL1*_ *-p14* ^*D122Y*^ *-myc-TEV* ^*234STOP*^ in pRS306K	[9]
pDS28	*ura3::kanMX::*P_*GAL1*_ *-p14* ^*D122Y*^ *-myc-TEV* ^*224STOP*^ in pRS306K	This study
pCR40	*ura3::kanMX::*P_*GAL1*_ *-p14* ^*D122Y*^ *-TEV* ^*R203G 234STOP*^ in pRS306K	This study
pCR29	*P*_*GAL1*_ *-GFP-p14* ^*D122Y*^ *-TEV* ^*234STOP*^ in pRS41N	This study
pCR39X20	*P*_*GAL1*_ *-GFP-p14* ^*D122Y*^ *-TEV* ^*234STOP*^ in pGREG566	This study
pGREG566	*P*_*GAL1*_ *-GFP::HIS3 kanMX URA3 ARS/CEN*	[43]

### Immunoblotting and calculation of pTEV2 protease cleavage efficiency

Immunoblotting experiments were performed as described using antibodies directed against GFP (Santa Cruz biotechnology, Santa Cruz, USA), tRFP (Biocat, Heidelberg, Germany), TEV protease (a kind gift of M. Ehrmann, (University of DuisburgEssen), tubulin (a kind gift of M. Knop, University of Heidelberg), and HRPO-coupled antibodies directed against mouse or rabbit IgG (Santa Cruz biotechnology, Santa Cruz, USA). The pTEV2 protease cleavage efficiency for the different amino acids at the P1' position was measured using immunoblots. The amount of full-length tester substrate was measured for each construct at the different time points and normalized to initial amounts (=100%). These values were normalized to proline (=no cleavage) to generate the graph.

### Quantitative fluorescence measurements

The RFP fluorescence was measured in yeast as follows. Cells were grown in liquid low fluorescence medium supplemented with 2% raffinose until the logarithmic growth phase was reached. Galactose (2% final concentration) was added to the cultures after removal of the first sample (t=0 hours) to induce protease production. Equal amounts of cells were taken at the indicated time points, treated with sodium azide (10 mM final concentration), and stored on ice until the end of the assay. Finally, samples were transferred to a black, flat-bottom 96-well microtiter plate (Greiner Bio-One, Germany) and the RFP fluorescence was measured with a microplate reader (Safire, TECAN, Crailsheim, Germany). Excitation conditions: 10 flashes of light with a wavelength of 555 nm; fluorescence was observed at a wavelength of 585 nm. Depletion efficiency was obtained from the mean curve by calculating the area above each curve. A depletion efficiency of 100% would be correlating to a curve with 0% RFP fluorescence at all time points, a depletion efficiency of 0% would be a curve with 100% RFP fluorescence at all time points. The higher the value for the depletion efficiency, the faster the construct is depleted from the cell.

### Generation of pTEV^+^ protease variants and red/white colony assay

Random mutagenesis of the pTEV^+^ protease was performed by standard procedure [40]. The mutagenic PCR was performed with taq polymerase in the presence of different manganese chloride concentrations (0 mM, 0.62 mM, and 1.25 mM) and a two-fold excess of dCTP and dTTP. In the first two rounds of mutagenesis, the plasmid pCT310 was used as template and the oligos p14end_for (TGTACTATAATGCCAACAGGG) and rec2-seq (GCGTGACATAACTAATTACATG) for PCR. Homologous recombination in yeast was used to clone the mutagenized PCR product into the *TEV protease* expression vector pCR29. A third round of mutagenesis was performed using the best performing clone of the first two screens (plasmid pCR30X2) as template and the oligos rec1_p14_tev (GAATTCGATATCAAGCTTATCGATACCGTCGACAATGGCGATGCAAGCGGCC) and rec2_p14_TEV234stop (GCGTGACATAACTAATTACATGACTCGAGGTCGACCTTACAATTGAGTCGCTTCC). Again, homologous recombination was used to clone the PCR product into plasmid pGREG566 [43] to obtain the *pTEV2 protease* expressing plasmid pCR39X20. The yeast transformants of each screening round were grown on non-inducing, selective medium (yeast extract peptone dextrose (YPD) containing 100 µg/ml Nourseothricin (pCR29) or 200 µg/ml Geneticin (pGREG566)). After two days at 30 °C, transformants were replicated on yeast extract peptone (YP) +galactose plates and incubated for two more days at 30 °C to induce the red colony phenotype. Development of dark-red colonies required additional incubation of the plates at 4 °C for several days. In total, about 1200 clones were screened in the red/white colony assay. After each round of mutagenesis, plasmids of positive clones were rescued in *E. coli*, retransformed into the yeast strain YCR6 and tested by a patch assay (as shown in Figure 2B) and immunoblotting for efficient Ade2-GFP-cODC1-TDegR-RFP depletion. Plasmids of confirmed positive clones were selected for sequencing. Positive clones obtained in the last round of mutagenesis were tested in a serial dilution growth test (Figure 2D).
